# The Analysis of OmpA and Rz/Rz1 of Lytic Bacteriophage from Surabaya, Indonesia

**DOI:** 10.1155/2021/7494144

**Published:** 2021-12-23

**Authors:** Tessa Sjahriani, Eddy Bagus Wasito, Wiwiek Tyasningsih

**Affiliations:** ^1^Doctoral Program, Faculty of Medicine, Universitas Airlangga, Dr. Moestopo Road No. 47, Surabaya 60285, Indonesia; ^2^Department of Microbiology, Faculty of Medicine, Universitas Malahayati, Pramuka Road No. 27, Bandar Lampung 35158, Indonesia; ^3^Department of Microbiology, Faculty of Medicine, Universitas Airlangga, Dr. Moestopo Road No. 47, Surabaya 60285, Indonesia; ^4^Department of Microbiology, Faculty of Veterinary Medicine, Universitas Airlangga, C Campus, Mulyorejo Road, Surabaya 60115, Indonesia

## Abstract

A good strategy to conquer the *Escherichia coli*-cause food-borne disease could be bacteriophages. Porins are a type of *β*-barrel proteins with diffuse channels and OmpA, which has a role in hydrophilic transport, is the most frequent porin in *E. coli*; it was also chosen as the potential receptor of the phage. And the Rz/Rz1 was engaged in the breakup of the host bacterial external membrane. This study aimed to analyze the amino acid of OmpA and Rz/Rz1 of lytic bacteriophage from Surabaya, Indonesia. This study employed a sample of 8 bacteriophages from the previous study. The OmpA analysis method was mass spectrometry. Rz/Rz1 was analyzed using PCR, DNA sequencing, Expasy Translation, and Expasy ProtParam. The result obtained 10% to 29% sequence coverage of OmpA, carrying the ligand-binding site. The Rz/Rz1 gene shares a high percentage of 97.04% to 98.89% identities with the *Siphoviridae* isolate ctTwQ4, partial genome, and *Myoviridae* isolate cthRA4, partial genome. The Mann–Whitney statistical tests indicate the significant differences between Alanine, Aspartate, Glycine, Proline, Serine (*p*=0.011), Asparagine, Cysteine (*p*=0.009), Isoleucine (*p*=0.043), Lysine (*p*=0.034), Methionine (*p*=0.001), Threonine (*p*=0.018), and Tryptophan (*p*=0.007) of OmpA and Rz/Rz1. The conclusion obtained from this study is the fact that OmpA acts as Phage 1, Phage 2, Phage 3, Phage 5, and Phage 6 receptors for its peptide composition comprising the ligand binding site, and Rz/Rz1 participates in host bacteria lysis.

## 1. Introduction

Bacteriophages are special viruses that invade bacterial cells [1]. Bacteriophages are a varied group of biological organisms that can be found in almost any environment where bacteria multiply [2, 3]. This virus has a life cycle in which it lyses cells to produce progeny in temperate environments [4, 5]. It is known, however different from antibiotic resistance, that bacteria have created mechanisms that defend them before phage [6], and the unconfirmed phage can promote the appearance of insensitive bacteriophage mutants and can lead to virulence transduction or antibiotic resistance to genes [7].

Bacteriophages often begin their infection process by attaching to the host cell surface via particular receptors on the cell surface [8]. As a result of infection, the bacteriophage's genetic material is injected into the cytoplasm of the bacterial cell. Bacteriophage infection is triggered by bacteriophage Receptor Binding Proteins (RBP) in the bacteriophage's tail end, as well as receptors associated with adsorption on the host cell surface, which have a structure that matches that of receptors on the host cell surface [9].

Bacteriophages must go through two procedures to start the replication process: seeking for and binding (adsorption) to the surface receptors on the bacterial cell wall and controlled perforation of the bacterial cell wall and delivery of viral DNA into the host cell cytoplasm [10]. The most significant element of the initial step of bacteriophage infection is at the matching tail/receptor, which allows the tail to construct a conduit across the bacterial cell wall to channel the viral genome into the host cell by further opening the head-to-tail connector for DNA [11]. The protrusion and insertion of the tail substructure into the bacterial cell wall are aided by activities that break down peptidoglycan and allow it to pass through the cell membrane barrier [12].

Gram-negative bacteria external membrane facilitates the transportation of proteins by barrel [13]. The *β*-barrel protein also acts as an enzyme for adhesion and pathogenicity. Porins are a class of *β*-barrel proteins with a channel on each monomer and are supplied with solution diffusion channels, homotrimers. OmpA [14] is the most prevalent porins of *E. coli*, which have a role in hydrophilic transportation. It was also chosen as the potential receptor of the phage [15–17].

The end of the penetrating tail tube can either directly merge with the cell membrane or engage with the host cell membrane channel, allowing bacteriophage DNA to be translocated barrier [12].

The Rz/Rz1 proteins are spanin complexes made up of o-spanins (outer membrane lipoproteins) and integral intracellular membrane proteins (i-spanins) [18]. The Rz/Rz1 gene was discovered in the lambdoid phage, in which state the DNA is known as a prophage and survives in the host genome without harming the host [19] and is involved in the degradation of the outer membrane of the host bacterium [20]. On the contrary, in the face of the threat posed by bacteriophages, bacteria do not stay defenseless, and microbiological testing by reducing host bacteria may be falsified by bacteriophages [21]. The amino acid composition between OmpA and Rz/Rz1 of lytic bacteriophage was investigated in this research.

## 2. Materials and Methods

### 2.1. Bacteriophage Preparation

A stock culture of bacteriophage *E. coli* O157 : H7 isolate was obtained from a prior study [22].

### 2.2. Bacteriophage Protein Isolation

Institute of Tropical Disease Laboratory, Universitas Airlangga, Surabaya, Indonesia, carried out bacteriophage protein isolation. The isolation phase of bacteriophage protein began with a suspension of 0.5 mL supplemented with 0.5 mL of 96% EtOH and then incubated at −20°C for 24 hours. 100 *μ*L of single-power SDS-PAGE sample lysis buffer was added to the protein precipitation following the separation of ethanol layer (Tris-HCl 62,5 m, 2% SDS, 6% 2-mercaptoethanol, 10% glycerol, 0,1% blue bromophenol, pH 6,8) and then heated to 10 minutes at 100°C [23].

### 2.3. Bacteriophage Protein Molecular Weight

SDS-PAGE was conducted in the Institute of Tropical Diseases, Universitas Airlangga, Surabaya, Indonesia, to assay bacteriophage protein molecular weight. The Pageruler Protein Marker with weight molecules of 10, 15, 25, 35, 40, 55, 70, 100, 130, and 180 kDa was employed as a marker. The gel is deposited on the bottom with a concentration of 15% polyacrylamide. Once the separating gel was firm, the collection gel concentration was 7.5% polyacrylamide. Proteins and markers were combined at a ratio of 4 : 1.

At 1000 rpm at room temperature, the mixture was centrifuged for 20 minutes and then boiled with boiling water for 5 minutes. Then, a 45 *μ*L volume was added to the well. A 20 mA of electric current and a 50 volts voltage for 3.5 hours were provided for electrophoresis. The gel is removed from a glass platform and then silver is stained; then, the electrophoresis is finished as the color from the base of gel was 0.5 cm to 1 cm [23].

### 2.4. Peptide Digestion

The digestion protocol was carried out by adding 10 mL of trypsin digest solution (12.5 mg/mL of trypsin, 25 mM of ammonium bicarbonate) to each gel piece and then incubated overnight at 37°C. The digested peptides were extracted by two incubations for 20 minutes with 10–20 L acetonitrile/ACN containing 1% Trifluoroacetic acid/TFA (adjusted to the size of the gel pieces). Three extractions were applied to large gel pieces. The collected extracts were dried by rotary evaporation and stored at −20°C for mass spectrometry analysis [24], located on the International Proteomics Laboratory, Australia.

### 2.5. Mass Spectrometry

The International Proteomics Laboratory, Australia, carried out analytics with mass spectrometry (MS). Following digestion of protein samples with trypsins and peptides by standard procedures [24], peptides were evaluated utilizing the high-performance liquid chromatography (HPLC) nano Prominence Shimadzu (Shimadzu) system with 5600 Triple TOF Mass Spectrometer (Sciex) by means of electrospray mass ionization spectrometry. In Agilent Zorbax 300SB-C18, 3.5 m (Agilent Technologies), tryptic peptides were loaded and separated by a linear gradient of water/acetonitrile/formic acid by 0.1% (v/v). The target proteins were evaluated to be identified using Mascot sequence matching software (Matrix Science) to match the UniProt database.

TOFTM analyzer was used to perform MS/MS (MDS SCIEX). A 2 ml standard dilution sample of the dry peptide was dissolved (30 : 70 ACN : water). The solution obtained was 1 : 10 diluted by matrix solution (CHCA, 10 mg/mL), and spots formed on the Opti-TOF 384 well stainless steel plate. The resultant solution was the first standard MS TOF that has been used for the analysis of sample spots. A second MS/MS was installed, and the 15 most intensive peaks of the first MS were focused on (excluding a peak known as trypsin). In MS mode, the laser is 400 times a fire point, and in MS/MS mode, it is 2000. The intensity of the laser is 2800 J (MS) and 3900 J (MS/MS). The weight of 400–4000 amu with a focal weight from 2100 amu.

TOF novo sequences are available automatically with the settings extensions DeNovo ExplorerTM 3.6 (Applied Biosystems) software:

  Enzyme: trypsin
  Fix modification: Carbamidomethyl (C)
  Mass tolerance: 0.2

The software produces a potential sequence automatically and awards a score between 0 and 100. The scoring shows the extent to which the theoretical fragmentation pattern corresponds to the fragmentation spectrum in the list of peaks. For additional analysis and main database search, the highest scoring order of each peptide was picked.

The de novo sequences obtained from MS/MS spectra were then BLAST matched through http://www.ncbi.nlm.nih.gov/BLAST. The search parameters for LC-MS/MS analysis on the 5600 TripleTOF mass spectrometer (AB Sciex) were as follows:

  Peptide tolerance: ±0.2
  Tol MS/MS: ±0.2
  Peptide load: 2 + 3 + dan 4+
  Massa: Monoisotopic
  Enzyme: Trypsin
  Miss cleavage: 1

After a search for BLAST, a scoring system was assigned to de novo peptides from each gel band. The three highest e-values are measured from the UniProt entry for every peptide which finds a homologous BLAST hit and the total points for this band are determined on the basis of the highest overall score [24].

### 2.6. Liquid Chromatography-Mass Spectrometry (LC-MS/MS)

Proteins were evaluated using the LC-MS/MS technology based on the peptide composition of the receptor site binding on each sample of eight types of bacteriophages. Fragmentation of the peptide pattern was used to identify the proteins. Ions with broad identity or approximation have been picked (ion cut-off scores altered to calculate the Mascot).

### 2.7. Bacteriophage DNA Extraction

Qiagen's manufacture's technique for bacteriophage DNA extraction was followed. 1 mL of each bacteriophage culture was centrifuged for 3 minutes at 13,000 rpm according to the Qiagen procedure for bacterial DNA extraction (Qiagen, Maryland, USA).

### 2.8. The Rz/Rz1 Gene Primer Design

The presence of the Rz/Rz1 gene was detected using a primer design created by the researcher using the Clone Manager Suite program with the NCBI accession number M65239.1 (1285 bp) nucleotide database, namely, CGTGATGTTGCTGCGCTCGATG (as many as 22 bp), with 59% GC and a melting temperature of 68°C, using forward in the sequence 982–1003 bp, namely, CG. With 52% GC and a melting temperature of 66°C, the reverse design uses a 1261–1283 bp sequence, CGATATGGGCAGCTCTATCTGCA (23 bp).

### 2.9. Amplification of Bacteriophage DNA Target

A 5 *μ*L template was used in a 12.5 *μ*L volume of 2x Intron master mix for PCR. 0.5 *μ*L of distilled water, 1 *μ*L of forward primer, and 1 *μ*L of reverse primer made up the PCR mixture. PCR was used to test the thermal cycler gene (Bio-Rad, Tokyo, Japan). The cycle program for denaturation, annealing, and extension temperatures is as follows: 1 cycle at 94°C for 5 minutes and 35 cycles at 94°C for 45 seconds, 30 seconds at 59°C, and 30 seconds at 72°C, with the last extension at 72°C for 5 minutes.

### 2.10. Agarose Gel

Agarose gel procedural referred to Taha KM [25].

### 2.11. Agarose Gel Electrophoresis

A 100-bp DNA ladder marker (NEXMark) was used to electrophorese a total of 4 *μ*L of PCR results on a 2% agarose gel. At 100 volts, electrophoresis was carried out for 30 minutes. The developing bands were visualized using a UV transilluminator.

### 2.12. Bacteriophage DNA Purification

The QIA quick PCR Purification Kit (Qiagen, Maryland, USA) was used to purify the PCR products. PE buffer was first mixed with 96% EtOH before usage. The PCR product was then mixed with PB buffer (5X the volume), and the sample was deposited in a QIA quick spin column in a 2 ml collecting tube, where it was diffused for 1 minute at 15,000 rpm. The upper section was removed and placed in a sterile 1.5 ml tube. EB buffer is also added to the center region of the membrane, which is left for 1 minute before being centrifuged for 1 minute.

### 2.13. Bacteriophage DNA Sequencing

Bacteriophage DNA sequencing was performed according to Thermo Fisher's instructions (Thermo Fisher Scientific, California, USA). DNA from purified PCR results is utilized as the template for the DNA sequencing process. The Sanger technique was used to sequence the data using the ABI Prism 310 sequencer. Labeling was done with a big dye. The amount of liquid used was 10 *μ*L. The samples were loaded into all wells of the ABI Prism 310 sequencer to read the results. The sequencing findings were examined on a display and printed using the Applied Biosystems® Genetic Analyzer, in a 96-well plate format, which appeared in graphic form after a few hours. 1^st^ Base DNA Sequencing Division, Malaysia, performed the DNA sequencing. The sequencing results were then analyzed by NCBI BLAST nucleotides, and then, a homologous BLAST hit was determined.

### 2.14. Statistical Analysis

The statistical analysis utilized in this study depended on the distribution of the data. The independent T-test was used for normal distribution data; if it was not, the analysis was done with the Mann–Whitney test [26].

## 3. Results and Discussion

### 3.1. Analysis of Bacteriophage Protein Molecular Weight

**Figure 1:**
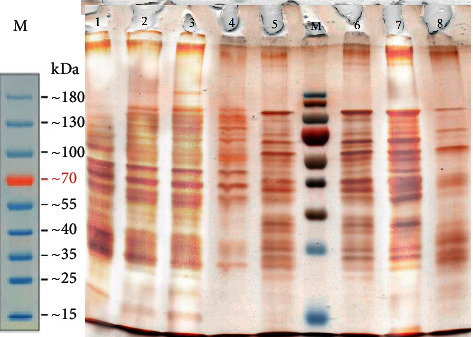
SDS-PAGE bacteriophage protein profile (*M* = Marker, 1 = Phage 1, 2 = Phage 2, 3 = Phage 3, 4 = Phage 4, 5 = Phage 5, 6 = Phage 6, 7 = Phage 7, 8 = Phage 8).

The protein profile of the SDS-PAGE bacteriophage investigation has demonstrated that the bacteriophage has multiple protein bands of various molecular weight dimensions as illustrated in Figure 1.

Bacteriophage protein molecular weight was discovered at 13.49 to 131.57 kDa, as reported in Table 1.

On prior investigations, OmpA was found at 30 to 35 kDa [27–34]. In order to be used in further analysis, bands in the region 23–40 kDa have been trimmed.

**Figure 2:**
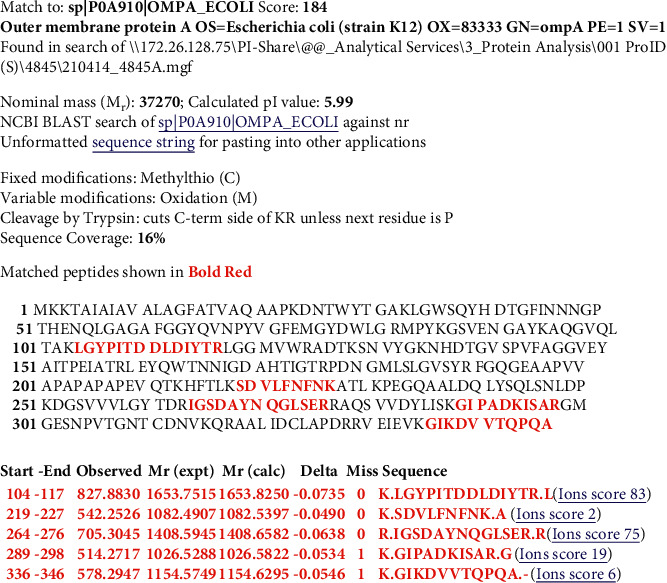
Phage 1 protein view of mass spectrometry.

**Figure 3:**
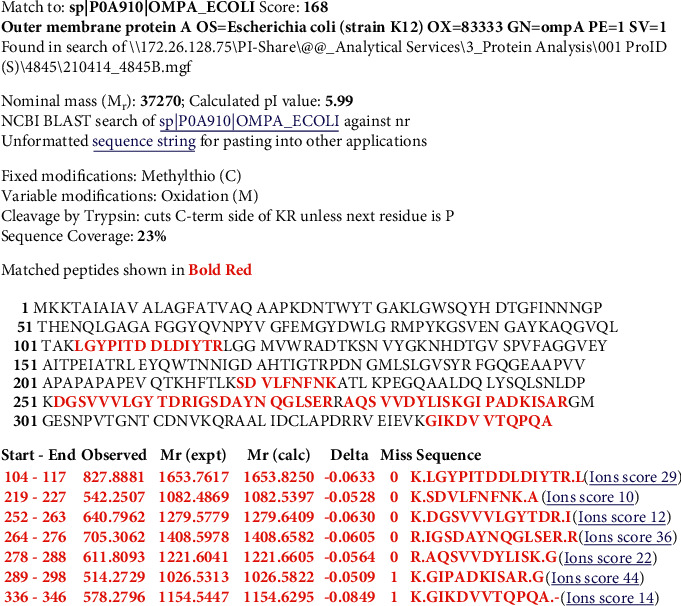
Phage 2 protein view of mass spectrometry.

**Figure 4:**
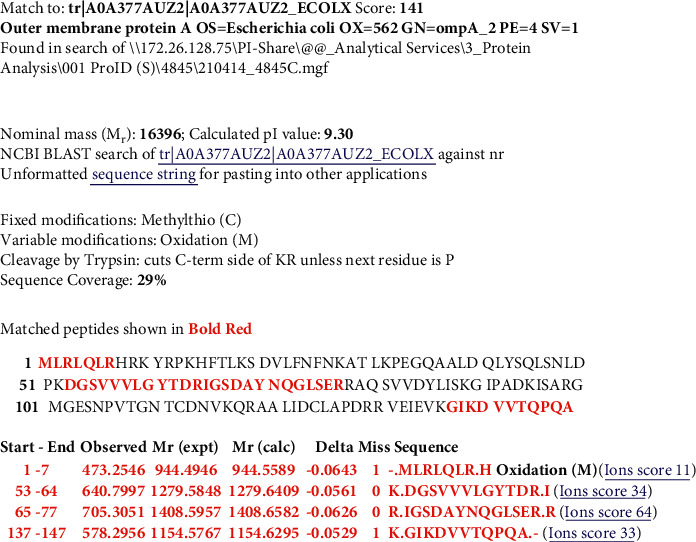
Phage 3 protein view of mass spectrometry.

**Figure 5:**
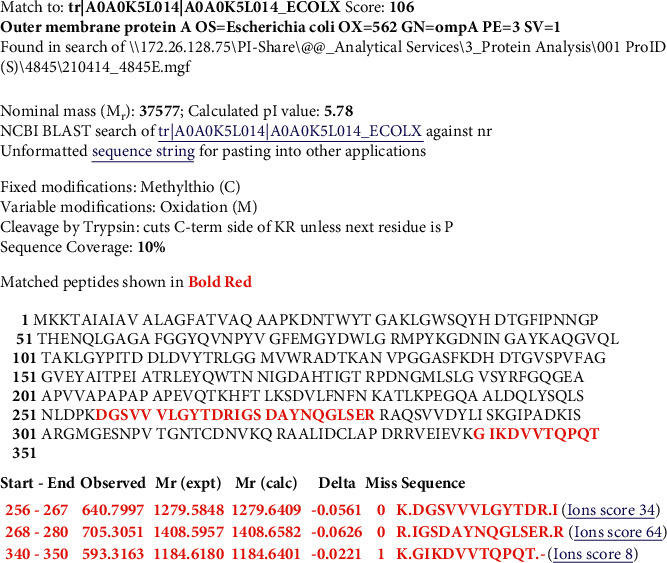
Phage 5 protein view of mass spectrometry.

Figures 2–5 show Mascot search results of protein view from the examination of mass spectrometry.

**Figure 6:**
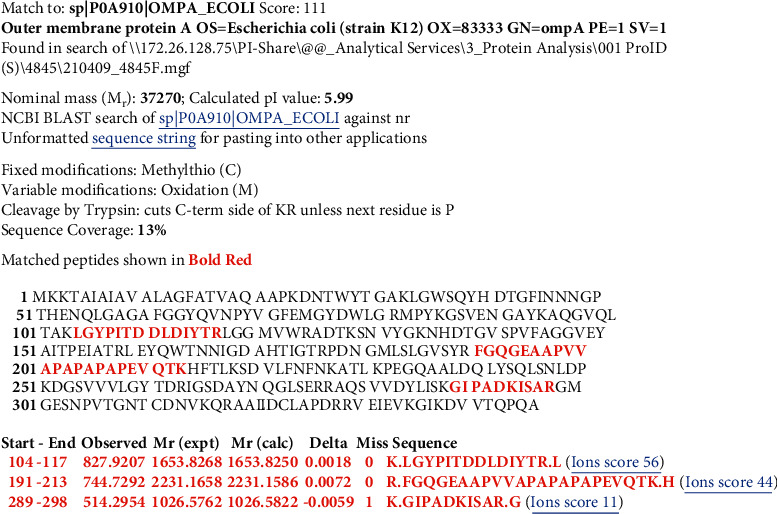
Phage 6 protein view of mass spectrometry.

The sequence covered 10 to 29% of the protein target, and peptides hit by LGYPITDLDIYTR, SDVLFNFNK, IGSDAYNQGLSER, GIPADCISAR, GIKDVVTQEPQA, DGSVVLGYTDR, AQSVVDYLISK, and FGQGEAPAPAPEVQTK are given in Figures 2–6, in the range of molecular weights 16,396 to 37,577 kDa. The score range was between 106 and 184, and the access code was issued by UniProt.  Table 2 displays the mass spectrometry assessment peptide view.

Phage 1's peptide hit on mass spectrometry was discovered to be comparable to Phage 2's and Phage 6's peptide hits, indicating that all three phages contain LGYPITDDLDIYTR and GIPADKISAR, presumably due to the same sample origin or location. With the difference between Phage 2 and Phage 1 in the peptide composition of DGSVVVLGYTDR and AQSVVDYLISK (in Phage 2), which may be caused by different sampling origins, the peptide in Phage 2 has the highest hit peptide composition, which is 6 peptides, and the least in Phage 6, with the difference between Phage 2 and Phage 1 in the peptide composition of DGSVVVLGYTDR and AQSVVDYLISK (in Phage 2). Differences in Phage 1 and Phage 2 with the peptide FGQGEAAPVVAPAPAPAPEVQTK in Phage 6 were also discovered, which could be related to different sample locations. Meanwhile, the SDVLFNFNK peptide was identified in Phages 1 and 2, and the IGSDAYNQGLSER peptide was discovered in Phage 1, Phage 2, Phage 3, and Phage 4, possibly as a result of the same sample location [22], affecting the respective peptide composition.

Both Phage 3 and Phage 5 have the same peptide hit, namely, DGSVVLGYTDR, IGSDAYNQGLSER, and GIKDVVTQPQA, despite the fact that they came from different samples [22]. The differences can be seen in the molecular weight, score, and UniProt BLAST, as well as different NCBI Blast accession numbers, where the IGSDAYNQGLSER peptide was also found in Phage 1 (with different samples).

The sequences obtained from MS were then BLAST matched through http://www.ncbi.nlm.nih.gov/BLAST; then, a homologous BLAST hit was determined. The NCBI BLAST protein has been assessable and recognized by OmpA as being the bacteriophage receptor in Phage 1, Phage 2, Phage 3, Phage 5, and Phage 6 in its peptide composition containing the ligand-binding site [15–17].

### 3.2. LC-MS/MS Analysis of Bacteriophage

The fragmentation of the bacteriophage peptide pattern of LC-MS/MS was seen hereinafter.

#### 3.2.1. LC-MS/MS Analysis of Phage 1

**Figure 7:**
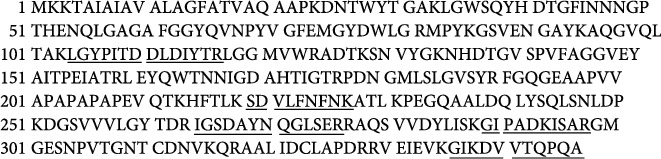
Mass spectrometry analysis of Phage 1.

LGYPITDDLDIYTR, SDVLFNFNK, IGSDAYNQGLSER, GIPADKISAR, and GIKDVVTQPQA have identified 16% of UniProt accession number sp|P0A910|OMPA_ECOLI as LC-MS/MS result from Phage 1 peptide sequences, as shown in Figure 7 (underlined).

#### 3.2.2. LC-MS/MS Analysis of Phage 2

**Figure 8:**
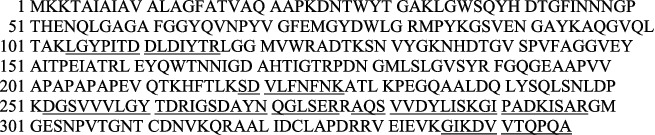
Mass spectrometry analysis of Phage 2.

In the following order of Figure 8, the findings of the LC-MS/MS of Phage 2 were detected in LGYPITDDLDIYTR, SDVLFNFNK, DGSVVVLGYTDR, IGSDAYNQGLSER, AQSVVDYLISK, GIPADKISAR, and GIKDVVTQPQA with a 23% match sequence with UniProt accession number sp|P0A910|OMPA_ECOLI (underlined).

#### 3.2.3. LC-MS/MS Analysis of Phage 3

**Figure 9:**
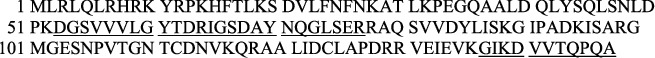
Mass spectrometry analysis of Phage 3.

Figure 9 shows the LC-MS/MS results of discovered peptides of Phage 3: DGSVVLGYTDR, IGSDAYNQGLSER, and GIKDVVTQPQA, which revealed a 29% match to UniProt accession number tr|A0A377AUZ2|A0A377AUZ2_ECOLX sequences (underlined).

#### 3.2.4. LC-MS/MS Analysis of Phage 5

**Figure 10:**
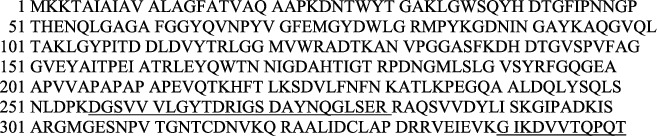
Mass spectrometry analysis of Phage 5.

The DGSVVLGYTDR, IGSDAYNQGLSER, and GIKDVVTQPQA MS findings of the identified Phage 5 peptides were solely 10% match to UniProt accession number tr|A0A0K5L014|A0A0K5L014_ECOLX, as shown in Figure 10 (underlined).

#### 3.2.5. LC-MS/MS Analysis of Phage 6

**Figure 11:**
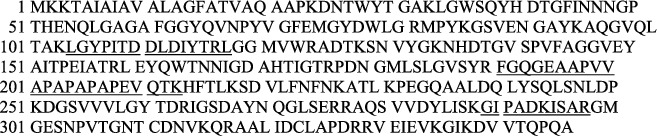
Mass spectrometry analysis of Phage 6.

As indicated in Figure 11, LC-MS/MS results of Phage 6 discovered peptides, LGYPITDDLDIYTR, FGQGEAAPVVAPAPAPAPEVQTK, and GIPADKISAR, exhibited a match of 29% Uniprot accession number sp|P0A910|OMPA_ECOLI sequences (underlined).

#### 3.2.6. LC-MS/MS Analysis of Phage 4, Phage 7, and Phage 8

In the meantime, sequence matches with OmpA in the NCBI sequence have not been discovered in Phage 4, Phage 7, and Phage 8.

RBPs are very specific, and so, the phage's host spectrum is primarily determined by them [35]. In order to start the infection process, RBPs must interact with their cell wall receptors [36]. Both lipopolysaccharide (LPS) and OMPs could be used as receptors by the bacteriophage [37]. RBPs allow phages to bind to a variety of cell surface components, including proteins, polysaccharides, LPS, and carbohydrate-binding moieties. Phages have a high level of functional plasticity due to genetic changes to RBPs and natural and laboratory-guided evolution, allowing them to adapt their activity and host range to a variety of hosts and environments [38–45]. In essence, the adaptability of a phage's RBP determines its capacity to survive.

RBPs have been used as therapeutic strategies to inhibit bacterial colonization due to their stability, particular binding nature, and affinity for certain carbohydrate-binding proteins [46]. RBPs bind to protein receptors on bacterial surfaces in the *B. subtilis* SPP1 phage, *B. anthracis* phage, and c2-type phages that infect *L. lactis* of Siphoviruses [47]. Many different molecular structures on bacteria's surfaces can operate as phage receptors, although their nature and location on the cell vary depending on the bacteria–phage interaction. LPS is also a frequent phage receptor in Gram-negative bacteria [48].

Depending on the type of ligand, bacteriophage receptors for bacteria can be categorized into three major groups. The first and most common category identifies carbohydrates in the cell wall or lipopolysaccharide. Proteins are recognized by the second group. The third group recognizes carbohydrate and protein-containing mixed receptors. In Gram-negative bacteria, bacteriophage receptors are recognized primarily by carbohydrates, LPS, and O-antigen. The second most prominent group of ligands is capsular polysaccharides, while protein ligands are centered on the Omp/Ton family. Gram-negative bacteriophages also carry specialized enzymes that cleave the host's O-antigens. Flagellins, pili, and mating pair structures are the most common protein ligands for this type of bacteriophages [48, 49].

Bacteriophages that recognize carbohydrate ligands must not only adhere to bacteria but also penetrate their cell walls to infect them. Gram-negative bacteria bacteriophages have specialized machinery to break down bacterial cell walls for this purpose. A carbohydrate hydrolase from the endolysin family capable of cleaving carbohydrate-carbohydrate bonds is the major protein tool [50]. The *Klebsiella pneumoniae* bacteriophage Kp32 tail tubular proteins TTPAgp31 and TTPAgp44 have recently been discovered to have glycolytic activity against biofilms and disaccharides [51]. P2 and TP901-1 phage are known to bind to a carbohydrate on the host's surface when they infect *L. lactis* [52]. In phage A118 (*Siphoviridae* group) for *L. monocytogenes*, rhamnose residues in wall teichoic acids serve as binding ligands for RBPs [53]. Characteristic sugar substituents in wall teichoic acid polymers, including N-acetylglucosamine (GlcNAc) and rhamnose (Rha), are hypothesized to serve as putative phage attachment receptors, i.e., binding of the matching RBP [54–56].

Most of these proteins, including *Bacillus* phage SPP1, are thought to bind to cell wall-associated carbohydrates like teichoic acids [57]. The D-glucose chain of teichoic acid on the *B. subtilis* surface, which serves as a receptor for phages SP2 and SP10, is another example [58]. Recognition and binding are exceedingly specific, and high affinity is essential for virus attachment to occur quickly and efficiently [59–61]. Phages can use many RBPs to attach to their target bacteria; therefore, finding one does not rule out the possibility of others [62–64]. Santos et al. investigated the use of wall teichoic acid as a *Staphylococcus* phage RBP [65]. Yan et al. looked into the utilization of the colicin A, targeting the outer membrane protein BtuB in *E. coli* phage [66], while others looked into FhuA as an RBP in *E. coli* phage [67] and phage T5 [52, 68]. LPS is also used as the key irreversible phage binding site in the model *E. coli* phage T7 [69]. R. D. Heselpoth, C. W. Euler, and R. Scuch examined the use of the bacteriocin pyocin in *P. aeruginosa* phage [70], as well as Ail in *Yersinia pestis* phage [71, 72]. It is envisaged that, by identifying the RBP, it may be possible to develop alternative narrow-spectrum antibiotics.

Monocins are discovered class of F-type bacteriocins produced by *L. monocytogenes*, a food-borne human pathogen. Monocins are similar to the tail structures of TP901-1 phages, unlike *P. aeruginosa* F-type bacteriocins, which are connected to lambda-like phage tails. The combination of the monocin was able to remove *L. monocytogenes* strains [73]. Aside from LPSs, RBPs have a depolymerase activity that can break down bacterial exopolysaccharides found in the capsule or biofilm matrix (LPS) [74–76]. The thick polysaccharide capsule was investigated as a receptor targeted by RBPs of *Klebsiella-*specific phages [75, 77].

### 3.3. Detection of the Bacteriophage Rz/Rz1 Gene

**Figure 12:**
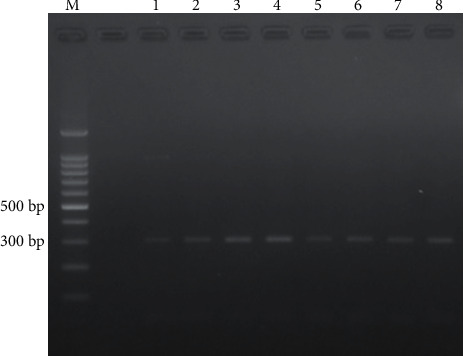
Detection of Rz/Rz1 gene (*M* = marker, 1–8 = number of bacteriophage samples).

The results of the Rz/Rz1 gene's molecular identification using the PCR technique are shown in Figure 12.

The Rz/Rz1 gene is likely to be present in the bacteriophage isolate at a location of roughly 300 bp. Furthermore, DNA sequencing was confirmed using PCR products in the eight putative bands of the Rz/Rz1 gene and then compared to sequence data from NCBI GenBank. The NCBI Blast program was used to match the sequencing results. The identity of proportional similarity and query cover is a factor in preference, so the selection is not necessarily dependent on a high total score, which is a low total score with the highest percentage, and the highest cover query can be used as a reference (according to the NCBI protocol in reading BLAST results). The following are the results of bacteriophage DNA sequencing confirmation.

#### 3.3.1. Rz/Rz1 Gene Sequence of Bacteriophage

With TPA Siphoviridae isolate ctTwQ4, partial genome coded BK029773.1, the Rz/Rz1 gene had a 98.11 to 98.89% homology, while TPA Myoviridae isolate cthRA4, partial genome, coded BK016886.1, shared 96.97 to 97.8% of its genome, respectively, as shown in Table 3.

The Bacteriophages 1, 2, 3, and 4 previously described as the bacteriophage family of Siphoviridae, while Bacteriophages 5, 6, 7, and 8 as the *Myoviridae* family [22], were found to have Rz/Rz1 sequences homology with TPA: *Siphoviridae* isolate ctTwQ4 partial genome, and TPA: *Myoviridae* isolate cthRA4, partial genome, from NCBI GenBank data.

Its selectivity against bacteria is the biggest benefit of the use of bacteriophages. In theory, if the amount of phage in the environment increases, the number of bacteria decreases [78]. On the contrary, in the face of the threat posed by bacteriophages, bacteria do not stay defenseless; microbiological testing by reducing host bacteria may be falsified by bacteriophages [21]. According to Berry [18], the Rz/Rz1 gene was discovered in the lambdoid phage, in which state the DNA is known as a prophage and survives in the host genome without harming the host [19] but was previously discovered in the lytic phage [22]. High temperatures, rich medium, and low infection multiplicity, all of which can stimulate lytic bacteriophages, can produce this [19, 79].

The process by which bacterial virus, known as bacteriophage or phage, mediates the transfer of DNA into bacteria is known as transduction. In one process, an infecting phage lyses the bacterial chromosome and replicates its own DNA using the host machinery. When bacterial DNA is accidentally incorporated into a phage head, this DNA can be passed to another bacterium in a following infection round. Under certain conditions, bacteriophage DNA can integrate itself into host cell chromosomes in the lysogenic pathway. In this state, the DNA is referred to as a prophage and remains in the host genome without damaging the host. The host is called a lysogen when a prophage is present. These prophages can enter the cycle when lysogen enters a state of stress [19].

Prophage transcription starts from the pL, pR, and pM promoters resulting in an “immediate early” transcript, expressing N and cro genes, producing N and cro proteins. Active PR performs transcription to produce mRNA. The N protein functions to link the RNA polymerase to a specific location of the newly transcribed mRNA. When RNA polymerase transcribes, it forms complexes with several host proteins [80]. Cro and cI are essential members that regulate the prophage's lytic excision [81], by self-regulating its promoter and limiting the expression of the other prophage genes [82].

Cro inhibits cI gene expression, whereas cI inhibits transcription from two important “lytic” promoters (pL and pR, which provide mRNAs for cro and other “lytic” genes, encoding proteins involved in all processes during phage progeny formation) while increasing its own expression via the pM promoter. As a result, the outcome of the cro-cI competition is critical in determining which of the two developmental pathways to pursue. Because no cI protein is present early after infection, another transcription regulator, the cII protein (whose gene is transcribed from pR), is a major player in this game. This protein activates the pE promoter, which is the second promoter for cI expression. As a result, cII activity determines whether cro or cI predominates [19].

Xis, int, Q, and genes for bacteriophage genome (OP) replication are still involved in “delayed early” transcription. Cro takes over the repressor site and prevents the PRM promoter from being synthesized (which is a lysogenic cycle promoter). By prompting bacterial DNA polymerase, proteins O and P start bacteriophage chromosomal replication [19]. Generalized transduction is named after the fact that any part of the bacterial chromosome can be transferred. Temperate or lysogenic phage and prophage contain phage integrated into the bacterial chromosome, allowing for specific transduction. Due to incomplete excision, a section of the chromosome near the phage's attachment site can be transferred. Some phages include virulence genes incorporated in their genomes, and when prophages depart their dormant condition in the chromosome and begin replicating during the lytic cycle, these virulence factors can be produced in large quantities [83].

**Figure 13:**
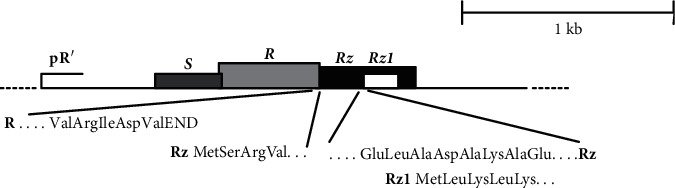
The Rz/Rz1 genes.

Rz/Rz1 is a product of phage genes transcribed from the *λ* late promoter PR [84]. The Rz/Rz1 gene is the membrane subunit of the spanin complex which is essential for the disruption of the outer membrane during phage lysis, as seen in Figure 13 [18, 85–87]. According to the Casjens and Hendrix study, similarity of the Rz/Rz1 gene of bacteriophage sequences was also identified in *Escherichia* phage lambda [88].

It may be possible for therapeutic strategies to reduce and inhibit bacterial activity and colonization with a variety of hosts by identifying the matching phage RBP as a result of natural and laboratory-guided evolution, as well as increasing the mechanism of bacteria–phage interaction binding on the ompA. For this aim, the Rz/Rz1 specialized machinery is activated to break down bacterial cell walls. This combination may be able to lyse and eliminate bacterial strains and lead to the development of new narrow-spectrum antibiotics.

### 3.4. Amino Acid Composition of OmpA and Rz/Rz1

Bacteriophages 1, 2, and 3 have been classified as the Siphoviridae bacteriophage family, while Bacteriophages 4, 5, and 6 were described as the Myoviridae family. The Rz/Rz1 amino acid was examined with Expasy Translation and Expasy ProtParam. Compared to OmpA, the amino acid composition of Rz/Rz1 was demonstrated in Table 4.

Alanine, Glycine, Valine, Asparagine, Leucine, Threonine, and Aspartate were the prominent amino-acids in the OmpA, where the percentage of Alanine, Asparagine, Glycine, Methionine, Phenylalanine, and Tryptophan in Bacteriophage 3 (Siphoviridae family) was low, but higher for Arginine, Aspartate, Isoleucine, Leucine, Lysine, and Valine. But Proline was found higher in Bacteriophage 4 (Siphoviridae family).

When compared with Rz/Rz1, the Proline, Serine, Leucine, Methionine, Valine, Alanine, Cysteine and Isoleucine were prominent, whereas Arginine, Cysteine, Tryptophan, and Valine are less in Bacteriophage 1 (Siphoviridae family), the same as Glutamine in Bacteriophage 2 (Myoviridae family) and Alanine and Isoleucine in Bacteriophage 5 (Myoviridae family). However, Histidine of Bacteriophage 1 (Siphoviridae family), Methionine, Proline, and Serine in Bacteriophage 5 (Siphoviridae family) were higher than the other sample. Phenylalanine and Tyrosine are not present in the Rz/Rz1 of bacteriophage.

### 3.5. Statistical Analysis

Alanine, Asparagine, Aspartate, Glycine, Histidine, Phenylalanine, Threonine, Tyrosine, and Valine in OmpA are more prominently comprised as shown in Table 5. The average in Rz/Rz1 seems to be higher: Arginine, Cysteine, Glutamine, Glutamate, Isoleucine, Leucine, Methionine, Proline, Serine, and Tryptophan, while Phenylalanine and Tyrosine are not in the Rz/Rz1 of bacteriophage. The descriptive group data was given in Table 5, according to the JASP 0.14.1.0.

The Shapiro-p Wilk's value was examined for data distribution analysis as indicated in Table 6.

Unnormal distribution data was revealed by the Shapiro–Wilk standard test. A nonparametric test based on the numerical variable for this investigation was employed with Mann–Whitney [29]. The Mann–Whitney test produced substantial results seen in Table 7 (underlined).

According to the Mann–Whitney test, the statistically significant differences between OmpA and Rz/Rz1 are between Alanine (*p*=0.011), Asparagine (*p*=0.009), Aspartate (*p*=0.011), Cysteine (*p*=0.009), Glycine (*p*=0.011), Isoleucine (*p*=0.043), Lysine (*p*=0.034), Methionine (*p*=0.001), Proline (*p*=0.011), Serine (*p*=0.011), Threonine (*p*=0.018), and Tryptophan (*p*=0.007).

## 4. Conclusions

OmpA acts as Phage 1, Phage 2, Phage 3, Phage 5, and Phage 6 receptor for its peptide composition comprising the ligand-binding site, and Rz/Rz1 participates in host bacteria lysis. The Mann–Whitney statistical tests indicate the significant differences between Alanine, Aspartate, Glycine, Proline, and Serine (*p*=0.011), Asparagine, Cysteine (*p*=0.009), Isoleucine (*p*=0.043), Lysine (*p*=0.034), Methionine (*p*=0.001), Threonine (*p*=0.018), and Tryptophan (*p*=0.007) of OmpA and Rz/Rz1 of lytic bacteriophage from Surabaya, Indonesia.

## Figures and Tables

**Table 1:** Protein molecular weight of bacteriophage.

No	Bacteriophage	Protein molecular weight (kDa)

1	Phage 1	72.63; 62.6; 53.96; 48.88; 40.09; 28.35; 25.68; 23.26; 19.08; 13.49
2	Phage 2	131.57; 62.6; 53.96; 48.87; 44.27; 29.79; 25.68; 21.06; 19.08; 16.44
3	Phage 3	131.57; 62.6; 53.96; 48.87; 44.27; 29.79; 25.68; 21.06; 19.08; 16.44
4	Phage 4	102.72; 93.03; 84.26; 72.63; 62.6; 53.96; 48.88; 28.35; 23.26
5	Phage 5	107.93; 84.26; 80.19; 72.63; 65.78; 59.58; 53.96; 48.88; 44.27; 40.09; 28.35; 25.68; 23.26
6	Phage 6	107.93; 84.26; 80.19; 72.63; 65.78; 59.58; 53.96; 48.88; 44.27; 40.09; 28.35; 25.68
7	Phage 7	107.93; 84.26; 80.19; 72.63; 65.78; 62.61; 59.58; 53.96; 48.88; 44.27; 40.09; 28.35; 25.68
8	Phage 8	107.93; 72.63; 53.96; 51.36; 28.35; 25.68; 23.26

**Table 2:** Peptide view of mass spectrometry.

Phage	Molecular weight (kDa)	Sequence coverage (%)	Peptide hit	Score	Uniprot accession number	NCBI Blast accession code

Phage 1	37,270	16	1. LGYPITDDLDIYTR	184	sp|P0A910|OMPA_ECOLI	STK07042.1
			2. SDVLFNFNK			
			3. IGSDAYNQGLSER			
			4. GIPADKISAR			
			5. GIKDVVTQPQA			

Phage 2	37,270	23	1. LGYPITDDLDIYTR	168	sp|P0A910|OMPA_ECOLI	STK07042.1
			2. SDVLFNFNK			
			3. DGSVVVLGYTDR			
			4. IGSDAYNQGLSER			
			5. AQSVVDYLISK			
			6. GIPADKISAR			

Phage 3	16,396	29	1. DGSVVVLGYTDR	141	tr|A0A377AUZ2|A0A377AUZ2_ECOLX	STL35309.1
			2. IGSDAYNQGLSER			
			3. GIKDVVTQPQA			

Phage 4	—	—	—	—	—	—
Phage 5	37,577	10	1. DGSVVVLGYTDR	106	tr|A0A0K5L014|A0A0K5L014_ECOLX	ABW72717.1
			2. IGSDAYNQGLSER			
			3. GIKDVVTQPQT			

Phage 6	37,270	13	1. LGYPITDDLDIYTRL	111	sp|P0A910|OMPA_ECOLI	STK07042.1
			2. FGQGEAAPVVAPAPAPAPEVQTK			
			3. GIPADKISAR			

Phage 7	—	—	—	—	—	—
Phage 8	—	—	—	—	—	—

**Table 3:** The Rz/Rz1 gene sequence percent identity.

Description	Percent identity								Accession
	Phage 1	Phage 2	Phage 3	Phage 4	Phage 5	Phage 6	Phage 7	Phage 8	

TPA: Siphoviridae isolate ctTwQ4, partial genome	98.90	98.15	98.89	98.54	98.11	98.89	98.89	98.89	BK029773.1
TPA: Myoviridae isolate cthRA4, partial genome	97.80	97.04	97.45	97.45	96.97	97.79	97.79	97.78	BK016886.1

**Table 4:** Amino acid composition of OmpA and Rz/Rz1.

Amino acid	Siphoviridae family						Myoviridae family			
	Phage 1		Phage 2		Phage 3		Phage 5		Phage 6	
	OmpA (%)	Rz/Rz1 (%)	OmpA (%)	Rz/Rz1 (%)	OmpA (%)	Rz/Rz1 (%)	OmpA (%)	Rz/Rz1 (%)	OmpA (%)	Rz/Rz1 (%)

Alanine	10.7	5.6	10.7	5.7	7.5	5.6	10.9	1.1	10.7	5.7
Arginine	3.8	6.7	3.8	4.5	7.5	4.5	3.7	5.7	3.8	4.6
Asparagine	5.5	3.3	5.5	3.4	4.8	3.4	5.1	3.4	5.5	3.4
Aspartate	6.4	4.4	6.4	2.3	8.2	2.2	6.9	1.1	6.4	2.3
Cysteine	0.6	4.4	0.6	5.7	1.4	5.6	0.6	5.7	0.6	5.7
Glutamine	4.9	4.4	4.9	6.8	6.1	5.6	4.9	4.6	4.9	4.6
Glutamate	3.8	2.2	3.8	4.5	3.4	5.6	3.4	2.3	3.8	5.7
Glycine	11.0	3.3	11.0	4.5	6.8	4.5	11.1	3.4	11.0	4.6
Histidine	1.4	2.2	1.4	1.1	1.4	1.1	1.4	0.0	1.4	1.1
Isoleucine	4.6	5.6	4.6	5.7	4.8	5.6	4.6	4.6	4.6	5.7
Leucine	6.6	7.8	6.6	8.0	10.2	6.7	6.6	8.0	6.6	6.9
Lysine	5.5	6.7	5.5	4.5	7.5	4.5	5.4	6.9	5.5	4.6
Methionine	1.7	6.7	1.7	6.8	1.44	6.7	1.7	9.2	1.7	6.9
Phenylalanine	2.6	0.0	2.6	0.0	2.0	0.0	2.9	0.0	2.6	0.0
Proline	5.5	12.2	5.5	12.5	4.8	13.5	6.0	13.8	5.5	12.6
Serine	4.6	12.2	4.6	11.4	6.8	11.2	4.3	12.6	4.6	11.5
Threonine	6.6	2.2	6.6	2.3	4.1	2.2	6.9	4.6	6.6	2.3
Tryptophan	1.4	3.3	1.4	3.4	0.0	3.4	1.4	3.4	1.4	3.4
Tyrosine	4.9	0.0	4.9	0.0	3.4	0.0	4.6	1.1	4.9	0.0
Valine	7.8	6.7	7.8	6.8	8.2	7.9	7.7	8.0	7.8	8.0

**Table 5:** Group descriptives.

	Group	*N*	Mean	SD	SE
Alanine	OmpA	5	10.100	1.456	0.651
	Rz/Rz1	5	4.740	2.035	0.910

Arginine	OmpA	5	4.520	1.666	0.745
	Rz/Rz1	5	5.200	0.980	0.438

Asparagine	OmpA	5	5.280	0.319	0.143
	Rz/Rz1	5	3.380	0.045	0.020

Aspartate	OmpA	5	6.860	0.780	0.349
	Rz/Rz1	5	2.460	1.197	0.535

Cysteine	OmpA	5	0.760	0.358	0.160
	Rz/Rz1	5	5.420	0.572	0.256

Glutamine	OmpA	5	5.140	0.537	0.240
	Rz/Rz1	5	5.200	1.010	0.452

Glutamate	OmpA	5	3.640	0.219	0.098
	Rz/Rz1	5	4.060	1.718	0.769

Glycine	OmpA	5	10.180	1.890	0.845
	Rz/Rz1	5	4.060	0.650	0.291

Histidine	OmpA	5	1.400	0.000	0.000
	Rz/Rz1	5	1.100	0.778	0.348

Isoleucine	OmpA	5	4.640	0.089	0.040
	Rz/Rz1	5	5.440	0.472	0.211

Leucine	OmpA	5	7.320	1.610	0.720
	Rz/Rz1	5	7.480	0.630	0.282
Lysine	OmpA	5	5.880	0.907	0.405
	Rz/Rz1	5	7.480	0.630	0.282

Methionine	OmpA	5	1.648	0.116	0.052
	Rz/Rz1	5	7.260	1.088	0.486

Phenylalanine	OmpA	5	2.540	0.329	0.147
	Rz/Rz1	5	0.000	0.000	0.000

Proline	OmpA	5	5.460	0.428	0.191
	Rz/Rz1	5	12.920	0.691	0.309

Serine	OmpA	5	4.980	1.026	0.459
	Rz/Rz1	5	11.780	0.593	0.265

Threonine	OmpA	5	6.160	1.159	0.518
	Rz/Rz1	5	2.740	1.041	0.465

Tryptophan	OmpA	5	1.120	0.626	0.280
	Rz/Rz1	5	3.380	0.045	0.020

Tyrosine	OmpA	5	4.540	0.650	0.291
	Rz/Rz1	5	0.000	0.000	0.000

Valine	OmpA	5	7.860	0.195	0.087
	Rz/Rz1	5	7.480	0.669	0.299

**Table 6:** Test of normality (Shapiro–Wilk).

		*W*	*p*
Alanine	OmpA	0.602	<0.001
	Rz/Rz1	0.573	<0.001

Arginine	OmpA	0.574	<0.001
	Rz/Rz1	0.799	0.080

Asparagine	OmpA	0.774	0.048
	Rz/Rz1	0.552	<0.001

Aspartate	OmpA	0.713	0.013
	Rz/Rz1	0.858	0.221

Cysteine	OmpA	0.552	<0.001
	Rz/Rz1	0.603	<0.001

Glutamine	OmpA	0.552	<0.001
	Rz/Rz1	0.827	0.131

Glutamate	OmpA	0.684	0.006
	Rz/Rz1	0.826	0.129

Glycine	OmpA	0.572	<0.001
	Rz/Rz1	0.754	0.032

Histidine	OmpA	NaN^a^	
	Rz/Rz1	0.883	0.325

Isoleucine	OmpA	0.552	<0.001
	Rz/Rz1	0.639	0.002

Leucine	OmpA	0.552	<0.001
	Rz/Rz1	0.803	0.086

Lysine	OmpA	0.592	<.001
	Rz/Rz1	0.803	0.086

Methionine	OmpA	0.552	<.001
	Rz/Rz1	0.618	0.001

Phenylalanine	OmpA	0.828	0.135
	Rz/Rz1	NaN^b^	

Proline	OmpA	0.869	0.262
	Rz/Rz1	0.892	0.369

Serine	OmpA	0.656	0.003
	Rz/Rz1	0.897	0.391

Threonine	OmpA	0.645	0.002
	Rz/Rz1	0.587	<0.001
Tryptophan	OmpA	0.552	<0.001
	Rz/Rz1	0.552	<0.001

Tyrosine	OmpA	0.676	0.005
	Rz/Rz1	NaN^c^	

Valine	OmpA	0.727	0.018
	Rz/Rz1	0.744	0.026

*Note.* Significant results suggest a deviation from normality. ^a^The variance in Histidine is equal to 0 after the grouping on the peptide. ^b^The variance in Phenylalanine is equal to 0 after the grouping on the peptide. ^c^The variance in Tyrosine is equal to 0 after the grouping on the peptide.

**Table 7:** Mann–Whitney test.

	Test	Statistic	df	*p*

Alanine	Student	4.789	8	0.001
	Mann–Whitney	25.000		0.011

Arginine	Student	−0.787	8	0.454
	Mann–Whitney	5.000		0.138

Asparagine	Student	13.174	8	<0.001
	Mann–Whitney	25.000		0.009

Aspartate	Student	6.887	8	<0.001
	Mann–Whitney	25.000		0.011

Cysteine	Student	−15.448	8	<0.001
	Mann–Whitney	0.000		0.009

Glutamine	Student	−0.117	8	0.910
	Mann–Whitney	16.000		0.517

Glutamate	Student	−0.542	8	0.602
	Mann–Whitney	10.000		0.671

Glycine	Student	6.847	8	<0.001
	Mann–Whitney	25.000		0.011

Histidine	Student	NaN^a^		—
	Mann–Whitney	NaN^a^		—

Isoleucine	Student	−3.722	8	0.006
	Mann–Whitney	3.000		0.043

Leucine	Student	−0.207	8	0.841
	Mann–Whitney	5.000		0.130

Lysine	Student	−3.240	8	0.012
	Mann–Whitney	2.000		0.034

Methionine	Student	−11.472	8	<0.001
	Mann–Whitney	0.000		0.009

Phenylalanine	Student	NaN^b^		—
	Mann–Whitney	NaN^b^		—

Proline	Student	−20.533	8	<0.001
	Mann–Whitney	0.000		0.011

Serine	Student	−12.832	8	<0.001
	Mann–Whitney	0.000		0.011

Threonine	Student	4.910	8	0.001
	Mann–Whitney	24.000		0.018

Tryptophan	Student	−8.051	8	<0.001
	Mann–Whitney	0.000		0.007

Tyrosine	Student	NaN^c^		
	Mann–Whitney	NaN^c^		

Valine	Student	1.220	8	0.257
	Mann–Whitney	13.000		1.000

^a^The variance in Histidine is equal to 0 after the grouping on the peptide. ^b^The variance in Phenylalanine is equal to 0 after the grouping on the peptide. ^c^The variance in Tyrosine is equal to 0 after the grouping on the peptide.

## Data Availability

The data used to support the findings of this study are included within the article.
